# Association between methylenetetrahydrofolate reductase (MTHFR) polymorphisms and lung cancer risk in Chinese people: An updated meta-analysis

**DOI:** 10.1097/MD.0000000000016037

**Published:** 2019-06-14

**Authors:** Rui Zhong, Qingling Chen, Xinyue Zhang, Mengmeng Li, Xin Zhang, Weihong Lin

**Affiliations:** a Department of Neurology; b Department of Hepatology, The First Hospital of Jilin University, Chang Chun, Ji Lin, China.

**Keywords:** CMTHFR C677T, lung cancer, MTHFR A1298C

## Abstract

**Background:**

The association between Methylenetetrahydrofolate Reductase (MTHFR) polymorphisms and lung cancer risk in Chinese people has been widely explored; however, the results remain controversial. Thus, we conducted a meta-analysis to investigate the association between MTHFR gene polymorphisms and susceptibility to lung cancer in Chinese people.

**Objective:**

We performed an updated meta-analysis to investigate the association between MTHFR gene polymorphisms and susceptibility to lung cancer in Chinese people.

**Methods:**

PubMed, EMBASE, WANFANG database, and CNKI were searched to collect eligible articles. The associations of MTHFR gene polymorphism with lung cancer risk were evaluated by calculating the pooled odds ratios (ORs) and the 95% confidence interval (CI). The dominant, recessive, heterozygous, homozygous, and allelic genetic models were used to calculate the combined ORs.

**Results:**

A total of 16 eligible studies were identified in the present meta-analysis. Evidence from the pooled results indicated a significant association between the MTHFR C677T polymorphism and lung cancer susceptibility in Chinese people under the dominant, recessive, homozygous and allelic genetic models (T vs C: OR = 1.252, 95% CI, 1.090–1.437; TT vs CC: OR = 1.741, 95% CI, 1.252–2.420. (TT + CT) vs CC: OR = 1.227, 95% CI, 1.030–1.426. TT vs (CT + CC): OR = 1.606, 95% CI, 1.207–2.137).

**Conclusion:**

The present updated meta-analysis demonstrated that the MTHFR C677T polymorphism was significantly associated with susceptibility to lung cancer in Chinese people. Additional case-control studies with large sample sizes are needed to validate our findings.

## 1 Introduction

Cancer has become the leading cause of death worldwide due to the growth and aging of the population.^[1]^ Lung cancer is the most common form of diagnosed cancer and has led to the most cancer deaths (approximately 18.4% of cancer deaths) in both sexes combined.^[2]^ Small cell lung cancer (SCLC) and non-small cell lung cancer (NSCLC) are the two main pathological types of lung cancer. It has been widely believed that both environmental risk factors, such as tobacco smoke exposure and genetic variation, are associated with the development of lung cancer.^[3–5]^

Evidence from recent studies has indicated that high serum folate levels are considered to have a protective effect against lung cancer.^[6,7]^ The findings of a recent meta-analysis also confirmed the protective role of serum folate against lung cancer.^[8]^ Folate metabolism participates in the process of DNA methylation and repair, which may prevent canceration. In addition, folate participates in one-carbon metabolism, and the importance of one-carbon metabolism in the high growth rate of cancer cells has been widely accepted.^[9]^ Methylenetetrahydrofolate Reductase (MTHFR) gene polymorphisms may regulate the expression of folate.^[10]^ By regulating folate expression, the MTHFR gene polymorphism is considered to be associated with susceptibility to lung cancer. C677T and A1298C are the two most common SNPs in the MTHFR gene, and these SNPs have been identified to influence plasma folate levels and MTHFR gene activity.^[11,12]^

The association between MTHFR gene polymorphisms and lung cancer risk has been widely studied; however, the results remain controversial. The results from a previous meta-analysis revealed a significant association between the MTHFR C677T polymorphism and an increased risk of lung cancer in Asian populations; however, the study did not find a significant association of the MTHFR A1298C polymorphism with susceptibility to lung cancer in Asians.^[13,14]^ Two meta-analyses^[15,16]^ were carried out to assess the association between MTHFR gene polymorphisms and lung cancer risk in Chinese people and draw a consistent conclusion. Those studies did not find a relationship between MTHFR gene polymorphisms and lung cancer risk in the overall population. With the aim of exploring these findings, we conducted an updated meta-analysis to investigate the association between MTHFR gene polymorphisms and susceptibility to lung cancer in Chinese people.

## 2 Materials and methods

Ethical approval was not necessary, and all analyses in the present study were based on previous published studies, thus no ethical approval and patient consent are required.

### 2.1 Literature search

This meta-analysis was completed according to the meta-analysis on the Genetic Association Studies Checklist. We conducted a systematic literature search with PubMed, EMBASE, WANFANG database and CNKI to collect eligible articles using the following key terms: (“lung cancer” OR “pulmonary cancer” OR “lung neoplasms”) AND (“MTHFR” OR “C677T” OR “A1298C”) (specific combination of keywords at PubMed) from inception to Nov. 10, 2018. The reference lists of included studies were also screened to identify additional articles. We restricted our search to case-control or cohort trials for meta-analysis. Only English and Chinese articles were involved.

### 2.2 Study selection

The trials included in the meta-analysis fulfilled the following inclusion criteria:

1. the study was limited to case-control or cohort trials;
2. the study could evaluate the relationship between MTHFR gene polymorphism and lung cancer risk; and
3. lung cancer was diagnosed based on standard criteria.
4. The study reported the gene type or allele frequencies in both cases and controls.

Accordingly, the exclusion criteria were as follows:

1. duplicates,
2. case reports, reviews, letters or meta-analysis were excluded
3. (3), as were studies with inadequate information and
4. studies with overlapping dates.

We first screened titles and abstracts and excluded studies that clearly did not fulfill the inclusion criteria. Studies that provisionally met the eligibility criteria were further assessed for eligibility by examination of the full text. Two reviewers (Zhong and Chen) independently checked the articles and resolved disagreements by discussion. Only the most relevant articles were included in the final analysis.

### 2.3 Quality assessment

Two reviewers independently assessed the quality of each included study in the meta-analysis according to the Newcastle-Ottawa scale. Trials with a score of at least 6 stars were of good quality and below 6 stars were of poor quality. We resolved disagreements by discussion, or the disagreements were judged by the third reviewer to ensure a consistent outcome.

### 2.4 Date abstraction

The following information was extracted from each study: first author, year of publication, province, area, study design and the number of cases and controls, allele frequencies, genotype distributions of cases and controls, and Hardy-Weinberg equilibrium among controls. When this information was incomplete, we checked the supplementary date and contacted the corresponding authors for the required data. Two reviewers independently extracted the main data from the selected studies.

### 2.5 Statistical analysis

The association of CMTHFR C677T and A1298C with lung cancer risk was evaluated by calculating the pooled odds ratios (ORs) and 95% confidence interval (CI). The dominant, recessive, heterozygous, homozygous and allelic genetic models were used to calculate the combined ORs. Heterogeneity was assessed by the *P* value of X^2^ and I^2^ statistics, and heterogeneity was significant if the i^2^ statistic was greater than 50% or if the *P* value was less than .1. We used a randomized effects model to pool the results for significant heterogeneity in our meta-analysis; otherwise, a fixed effects model was applied. To explore the potential source of heterogeneity, subgroup analysis was carried out based on area (North China vs South China). In addition, sensitivity analysis, by removing one study at a time, was conducted to evaluate the stability of the results. We performed Begg test and Egger test to assess publication bias (*P* < .05 was considered statistically significant). All statistical analyses were conducted with STATA 12.0 software. A *P* value of <.05 was considered statistical significance.

## 3 Results

### 3.1 Study selection

**Figure 1:**
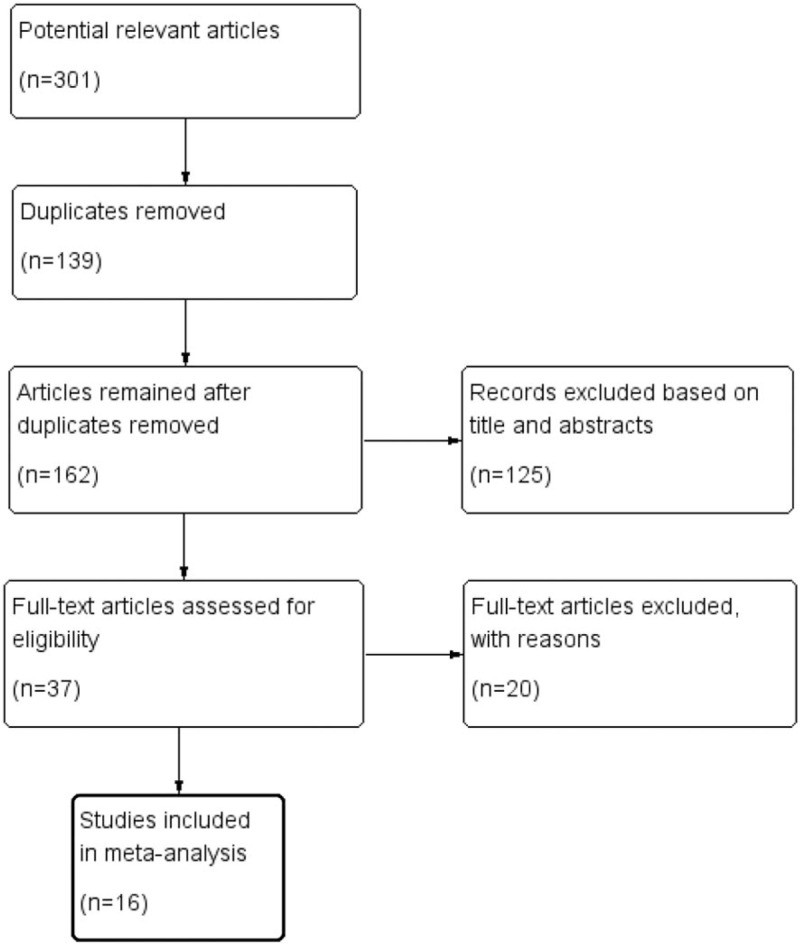
The process of identification of the studies.

A total of 301 potentially relevant articles were identified from initial database searching, of which 139 publications were removed due to duplicates; 162 studies remained after duplicates were excluded. Then, we excluded 125 articles that were obviously irrelevant based on titles and abstracts, and the remaining 37 articles were assessed in detail for full text. After full text assessment, we eventually identified 16^[17–32]^ studies that could be used for the meta-analysis. A flowchart of the process used for identification of studies is presented in Figure 1.

### 3.2 Characteristics of the studies and quality assessment

Eventually, 16 case-control studies with a total of 9294 individuals (4023 cases and 5270 controls) met our inclusion criteria and were included in our meta-analysis. Sixteen studies assessed the association between CMTHFR C677T polymorphisms and lung cancer susceptibility, and 8 studies investigated CMTHFR A1298C and lung cancer risk. Of the included studies, 9 were carried out in South China, and 7 were performed in North China. The characteristics of the included articles were summarized in Table 1. The quality of 16 trials was evaluated using the Newcastle-Ottawa scale, and the results are also shown in Table 1. Two studies received a score of 8, 9 studies scored 7, and 5 studies scored 6, indicating high quality. In addition, the genotype and allele distribution of the CMTHFR gene among cases and controls are listed in Table 2. For CMTHFR C677T, genotype values in the control group of 13 studies were in agreement with HWE. For CMTHFR A1298C, the genotype distribution of the controls in 1 study was not in compliance with the HWE.

**Table 1:**
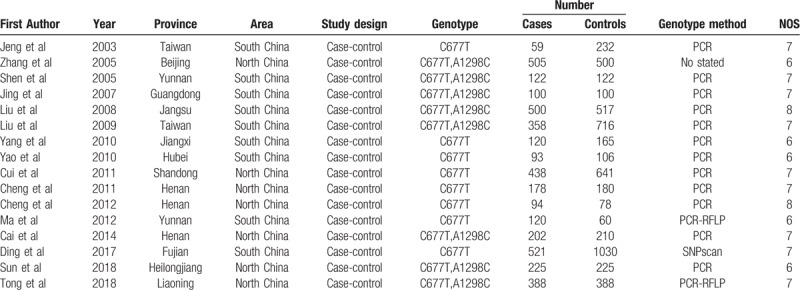
The characteristics of the included studies.

**Table 2:**
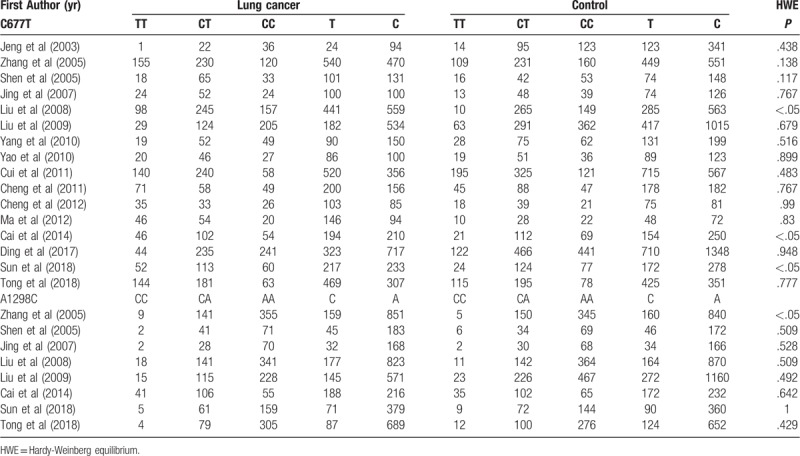
CMTHFR (C677T and A1298C) genotype and allele distribution among lung cancer cases and controls.

### 3.3 Meta-analysis of CMTHFR C677T polymorphisms

**Figure 2:**
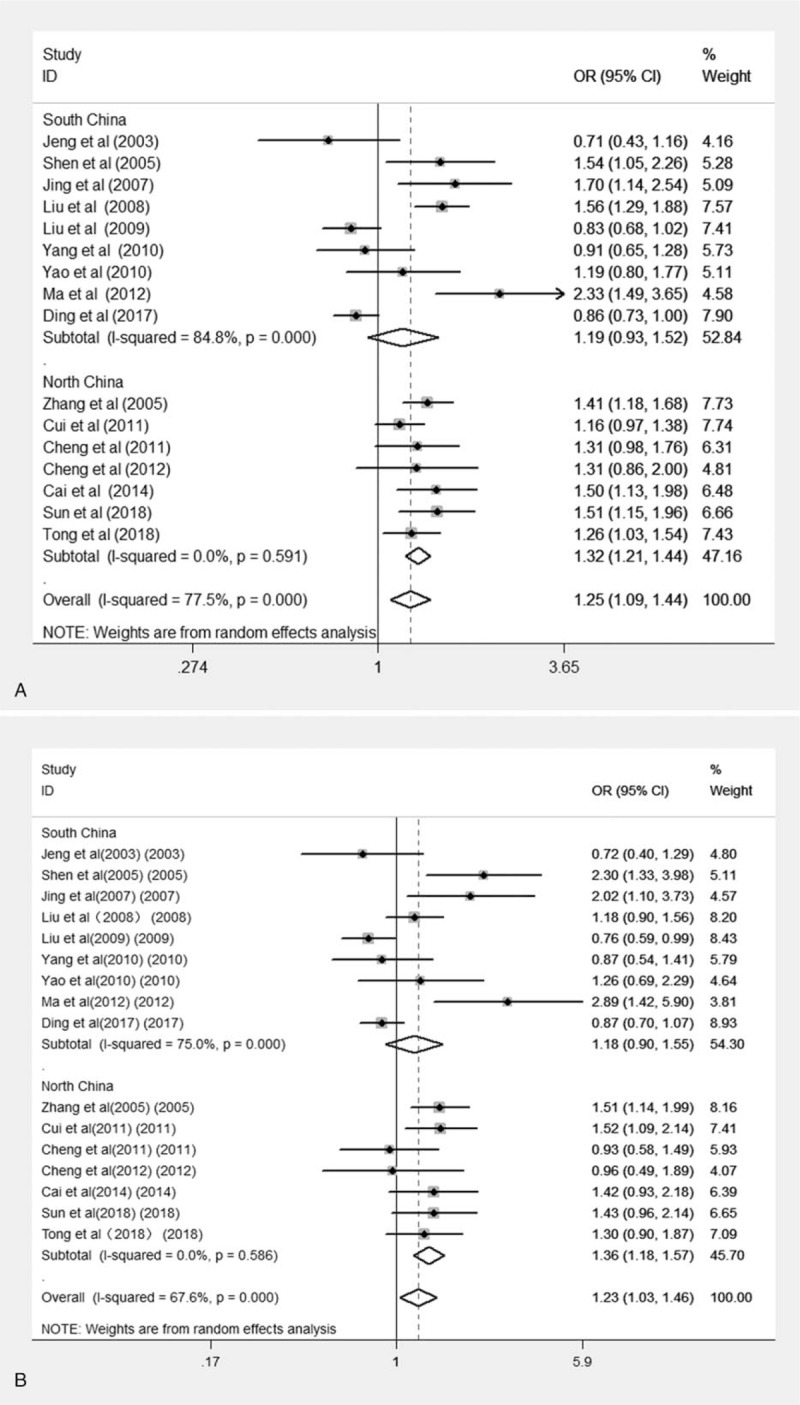
A Forest plot of the association between the CMTHFR C677T polymorphism (T vs C) and lung cancer susceptibility. B Forest plot of the association between the CMTHFR C677T polymorphism (TT + CT vs CC) and lung cancer susceptibility.

**Figure 3:**
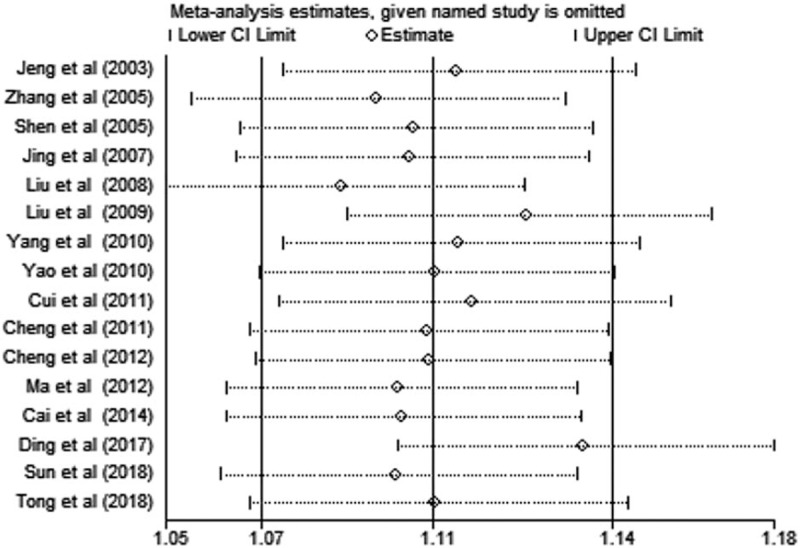
Sensitivity analyses of the association between the CMTHFR C677T polymorphism (T vs C) and lung cancer susceptibility.

**Figure 4:**
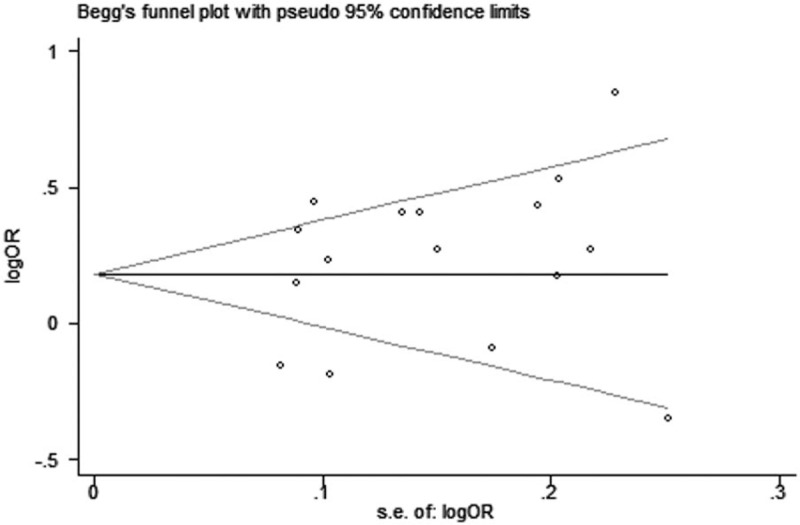
Funnel plot of the association between the CMTHFR C677T polymorphism (T vs C) and lung cancer susceptibility.

Sixteen case-control studies with a total of 4023 cases and 5270 controls assessed the association of CMTHFR C677T polymorphisms with susceptibility to lung cancer. The combined analysis revealed that there was a significant relationship between CMTHFR C677T polymorphisms and susceptibility to lung cancer in Chinese people overall under dominant, recessive, homozygous and allelic genetic models (T vs C: OR = 1.252, 95% CI, 1.090–1.437; TT vs CC: OR = 1.741, 95% CI, 1.252–2.420. (TT + CT) vs CC: OR = 1.227, 95% CI, 1.030–1.426. TT vs (CT + CC): OR = 1.606, 95% CI, 1.207–2.137. In subanalysis based on the area of China (North China vs South China), we observed similar results in the North China population under all genetic models (T vs C: OR = 1.321, 95% CI, 1.212–1.440; TT vs CC: OR = 1.796, 95% CI, 1.505–2.145; CT vs CC: OR = 1.176, 95% CI, 1.009–1.371; (TT + CT) vs CC: OR = 1.362, 95% CI, 1.179–1.575. TT vs (CT + CC): OR = 1.677, 95% CI, 1.316–2.138). However, we did not find any association between CMTHFR C677T polymorphisms and lung cancer risk in the South China population under all genetic models. The results of the meta-analysis of CMTHFR C677T polymorphisms are shown in Table 3 and Figure 2. We performed sensitivity analysis to assess the stability of the results, and there was no significant change in the overall results by removing each study (Fig. 3). We conducted Begg test and Egger test to assess the publication bias of the included studies. The results are shown in Table 3, and no publication bias was observed. A funnel plot for the association between MTHFR C677T and lung cancer risk is shown in Figure 4.

**Table 3:**
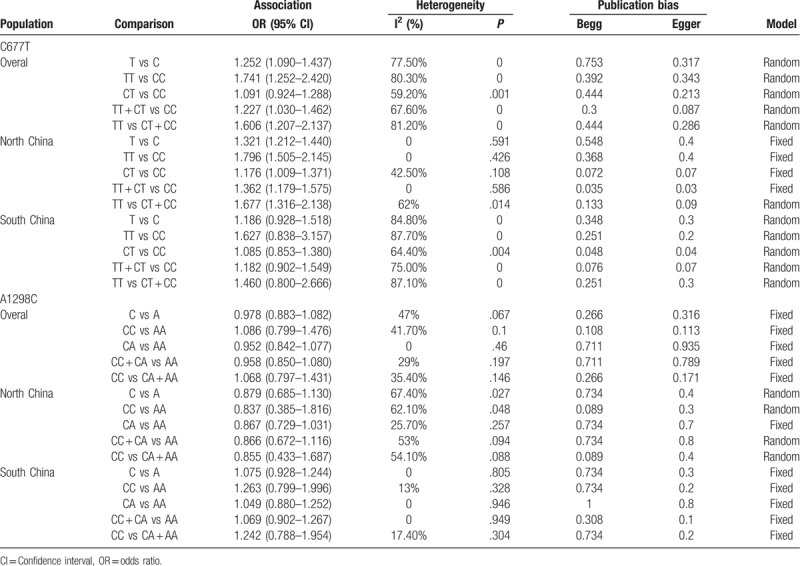
Meta-analysis of CMTHFR gene polymorphisms and susceptibility to lung cancer.

### 3.4 Meta-analysis of CMTHFR A1298C polymorphisms

Eight studies including 2400 lung cancer cases and 2778 controls evaluated the relationship between the MTHFR A1298C polymorphism and lung cancer susceptibility. We did not find a significant association between the MTHFR A1298C polymorphism and lung cancer risk in the overall Chinese population with any of the genetic models. We performed a subgroup meta-analysis by area of China and did not observe any association of the MTHFR A1298C polymorphism with lung cancer risk in either the North China or South China populations under all genetic models. The results are shown in Table 3.

Sensitivity analyses revealed the stability of the results. We did not observe publication bias (*P* > .05).

## 4 Discussion

The findings of our meta-analysis indicated that the CMTHFR C677T polymorphism was significantly associated with an increased risk of lung cancer in the overall Chinese and North China populations. However, we did not find an association between the MTHFR A1298C polymorphism and lung cancer susceptibility in Chinese people. In other words, the individuals with the TT genotype in the CMTHFR C677T gene had a significantly higher risk for developing lung cancer compared with those bearing the CC genotype, and carriage of the T allele in the CMTHFR C677T gene increased the susceptibility to lung cancer.

The association between CMTHFR gene polymorphisms and lung cancer susceptibility has been widely studied, especially for the CMTHFR C677T polymorphism. However, definite results cannot be reached. Shen et al^[33]^ first reported the relationship between the CMTHFR C677T polymorphism and lung cancer risk in their case-control study and did not find an association between the CMTHFR C677T polymorphism and lung cancer risk. Their conclusion was supported by several subsequent studies.^[23,24,34]^ However, Liu et al stated the opposite in their findings, and the results of their study revealed a significant association of the CMTHFR C677T polymorphism with an increased risk of lung cancer. Some subsequent studies also replicated this result.^[25,28,29,32]^ Previously, several meta-analyses were performed to evaluate the association of CMTHFR gene polymorphisms with lung cancer susceptibility. A recent meta-analysis^[13]^ revealed that the CMTHFR C677T polymorphism is significantly associated with an increased risk of lung cancer in Asian and overall populations but not in Caucasian populations. However, the meta-analysis did not find a significant association between the CMTHFR A1298C polymorphism and lung cancer risk. With the aim of assessing the relationship between CMTHFR gene polymorphisms and lung cancer susceptibility in Chinese people, 2 previous meta-analyses^[15,16]^ based on Chinese people were carried out; however, they did not observe a significant association of CMTHFR gene polymorphisms with lung cancer risk in the overall population, which was inconsistent with the results of other meta-analyses.^[14]^

Recently, more case-control studies on this topic were performed to test previous findings. Thus, we conducted an updated meta-analysis to assess the association between CMTHFR gene polymorphisms and lung cancer susceptibility in Chinese people. We included 16 eligible articles in the meta-analysis, of which 16 related to the C677T polymorphism and 8 related to the A1298C polymorphism. In the present meta-analysis, we found that the T allele in CMTHFR C677T, compared with the C allele, led to an increased risk of lung cancer in Chinese people. In addition, the results revealed that the TT genotype of CMTHFR C677T was indicative of a higher risk of lung cancer than the CC genotype. The significant association between the C677T polymorphism and susceptibility to lung cancer was also shown in dominant and recessive models. Our findings completely differ from the results of a previous meta-analysis.^[15,16]^ The different results may be attributed to the sample size and genetic backgrounds in different areas of China. As is well known, the association between gene SNPs and disorders is largely dependent on the sample size. Our meta-analysis thus provides a more accurate conclusion based on sample size. In the present meta-analysis, subgroup analysis based on areas of China indicated that there was a significant association between the CMTHFR C677T polymorphism and an increased risk of lung cancer in the North China population but not in the South China population. However, no association was observed between the MTHFR A1298C polymorphism and lung cancer susceptibility in both the North China and South China populations. A recent case-control study was performed in a female Chinese population, and a significant association between the MTHFR A1298C polymorphism and a decreased risk of lung cancer was observed in Chinese women.^[32]^ However, we failed to perform a subgroup analysis by gender due to a lack of detailed information on gender. For the meta-analysis, the CMTHFR C677T polymorphism and heterogeneity decreased after subgroup analysis in the North China population in dominant, heterozygous, homozygous and allelic genetic models; however, heterogeneity persisted in the South China population in all genetic models. Thus, the different areas of China may lead to a high degree of heterogeneity in the North China population. Furthermore, sensitivity analyses indicated that the results were statistically robust, and no publication bias was found, indicating the stability of the results of our meta-analysis. Our findings were consistent with the results of a previous meta-analysis based on the East Asian population by Zhang et al.^[14]^ In contrast to our study based on Chinese people, the meta-analysis by Zhang et al was conducted on East Asian populations, including Chinese, Japanese, and Korean peoples.

Several limitations of the meta-analysis should be acknowledged. First, a meta-analysis may be biased when the literature search fails to identify all relevant studies. However, access to unpublished articles remains difficult, which might be a potential limitation of our study. Only English and Chinese studies were included in our meta-analysis, which may have caused publication bias. Begg test and Egger test were used to investigate the publication bias. However, no significant publication bias was observed by Begg test or Egger test, indicating the stability of the results. Second, all analyses were based primarily on unadjusted ORs, and confounding factors were controlled. In addition, we were unable to assess all gene-to-environment and gene-to-gene interactions. Third, subgroup analyses based on gender, smoking status or type of lung cancer could not be conducted. Thus, more case-control studies with large sample sizes and detailed characteristics are needed. Finally, significant heterogeneity existed in several genetic models, and we did not find the source of the heterogeneity through subgroup analysis and sensitivity analyses.

In conclusion, our meta-analysis found that the CMTHFR C677T polymorphism was associated with a high risk of lung cancer in the overall Chinese and North Chinese populations. We found that the T allele and TT genotype lead to an increased risk of lung cancer in Chinese people. In addition, we did not observe an association between the MTHFR A1298C polymorphism and lung cancer susceptibility. Further case-control studies with large sample sizes are needed to validate our findings.

## Author contributions

**Conceptualization:** Rui Zhong, Mengmeng Li, Xin Zhang.

**Data curation:** Rui Zhong, Qingling Chen.

**Formal analysis:** Rui Zhong.

**Funding acquisition:** Rui Zhong.

**Investigation:** Rui Zhong, Qingling Chen, Xin Zhang.

**Methodology:** Rui Zhong, Qingling Chen, Xinyue Zhang.

**Project administration:** Rui Zhong, Mengmeng Li.

**Resources:** Rui Zhong, Qingling Chen, Mengmeng Li.

**Software:** Rui Zhong, Qingling Chen, Xinyue Zhang, Mengmeng Li, Xin Zhang, Weihong Lin.

**Supervision:** Rui Zhong, Weihong Lin.

**Validation:** Rui Zhong, Qingling Chen, Xinyue Zhang.

**Visualization:** Rui Zhong, Xin Zhang.

**Writing – original draft:** Rui Zhong, Qingling Chen, Weihong Lin.

**Writing – review & editing:** Rui Zhong, Xinyue Zhang, Weihong Lin.
